# Protective effects of rolipram on endotoxic cardiac dysfunction via inhibition of the inflammatory response in cardiac fibroblasts

**DOI:** 10.1186/s12872-020-01529-7

**Published:** 2020-05-24

**Authors:** Jingjing Ji, Zhifeng Liu, Xinxin Hong, Zheying Liu, Jinghua Gao, Jinghua Liu

**Affiliations:** 1 Guangdong Provincial Key Laboratory of Proteomics; School of Basic Medical Sciences, Southern Medical University, Guangzhou, 510515 China; 2 Department of Critical Care Medicine, General Hospital of Southern Theatre Command of PLA, Guangzhou, 510010 China; 3 Key Laboratory of Hot Zone Trauma Care and Tissue Repair of PLA, General Hospital of Southern Theatre Command of PLA, Guangzhou, 510010 China; 4 Guangdong Provincial Key Laboratory of Molecular Oncologic Pathology, Southern Medical University, Guangzhou, 510515 China; 5 Graduate School, Guangzhou University of Chinese Medicine, Guangzhou, 510006 China

**Keywords:** Sepsis induced cardiomyopathy, Rolipram, Inflammatory mediators, Cardiac fibroblasts, Dual specificity phosphatase 1

## Abstract

**Background:**

Cardiac fibroblasts, regarded as the immunomodulatory hub of the heart, have been thought to play an important role during sepsis-induced cardiomyopathy (SIC). However, the detailed molecular mechanism and targeted therapies for SIC are still lacking. Therefore, we sought to investigate the likely protective effects of rolipram, an anti-inflammatory drug, on lipopolysaccharide (LPS)-stimulated inflammatory responses in cardiac fibroblasts and on cardiac dysfunction in endotoxic mice.

**Method:**

Cardiac fibroblasts were isolated and stimulated with 1 μg/ml LPS for 6 h, and 10 μmol/l rolipram was administered for 1 h before LPS stimulation. mRNA levels of tumor necrosis factor-α (TNF-α), interleukin-6 (IL-6) and interleukin-1β (IL-1β) in fibroblasts and their protein concentrations in supernatant were measured with real-time PCR (rt-PCR) and enzyme-linked immunosorbent assay, respectively. The expression of dual specificity phosphatase 1 (DUSP1), an endogenous negative regulator that inactivates MAPK-mediated inflammatory pathways, was also measured by rt-PCR and western blotting. DUSP1-targeted small interfering RNA (siRNA) was used to examine the specific role of DUSP1. To evaluate the role of rolipram in vivo, an endotoxic mouse model was established by intraperitoneal injection of 15 mg/kg LPS, and 10 mg/kg rolipram was intraperitoneally injected 1 h before LPS injection. mRNA and protein levels of inflammatory cytokines and DUSP1 in heart, inflammatory cell infiltration and cardiac function were all examined at 6 h after LPS injection.

**Results:**

The results showed that LPS could increase the expression and secretion of inflammatory cytokines and decrease the transcription and expression of DUSP1 in cardiac fibroblasts. However, rolipram pretreatment significantly reversed the LPS-induced downregulation of DUSP1 and inhibited LPS-induced upregulation and secretion of TNF-α and IL-6 but not IL-1β. Moreover, DUSP1-targeted siRNA experiments indicated that the protective effect of rolipram on inflammatory response was specific dependent on DUSP1 expression. Moreover, rolipram could further reduce inflammatory cell infiltration scores as shown by pathological analysis and increase the ejection fraction (EF) detected with echocardiography in the hearts of endotoxic mice.

**Conclusions:**

Rolipram could improve endotoxin-induced cardiac dysfunction by upregulating DUSP1 expression to inhibit the inflammatory response in cardiac fibroblasts, which may be a potential treatment for SIC.

## Background

According to the Sepsis new definition, life-threatening organ dysfunction caused by a dysregulated host response to infection, Sequential Organ Failure Assessment (SOFA) score was employed as the diagnostic criteria, replacing the prevailing systemic inflammatory response syndrome (SIRS) criteria [1]. This change emphasizes the life-threatening organ dysfunction in sepsis, indicating that researchers and physicians should not only focus on the inflammatory response but also pay more attention to organ protection. The heart is one of the most frequently affected organs in sepsis. Sepsis-induced cardiomyopathy (SIC) has been reported to be present in more than 40–50% of cases of sepsis [2, 3]. Previous studies reported that the mortality of septic patients ranged from 28 to 48.4% [4, 5] and a higher mortality was observed in patients with observed cardiovascular dysfunction, with an odds ratio of 2.78 [6]. At present, no formalized or consensus definition of SIC exists. Generally, SIC is often diagnosed when some acute perturbation in cardiac function, systolic function or diastolic function exists in the setting of sepsis [7]. SIC has been recognized for 40 years [8], but its mechanism and process are still not well understood.

Over recent decades, a number of experimental and clinical studies have suggested possible causative mechanisms for progressive cardiac dysfunction, including disturbed coronary blood flow, cardiomyocyte apoptosis, effects of myocardial depressant factor (MDF), nitric oxide and reactive oxygen species, mitochondrial dysfunction, and calcium trafficking [9, 10]. Among these hypotheses, MDF and nitric oxide seemed to have larger effects on cardiac dysfunction in septic states. In 1985, Parrillo et al proposed that myocardial depressant substances existed in septic patients and that these depressant substances were the pathophysiologic factors that induced cardiomyopathy during sepsis [11]. Subsequently, some studies found that MDF was likely to be an endotoxin, a cell wall component of gram-negative bacteria. With more in-depth research in this field, further studies revealed that inflammatory cytokines had comparable effects to those of MDF. Of these cytokines, tumor necrosis factor-α (TNF-α), interleukin-6 (IL-6) and interleukin-1β (IL-1β), which were produced excessively in the early stage of sepsis, have been found to have potential depressive effects on cardiac function [10, 12].

Cardiac fibroblasts can respond to various types of external stimuli and are regarded as the immunomodulatory hub of the heart. Under physiological conditions, cardiac fibroblasts play important roles, such as electric isolation and/or conductance in different compartments and chemokine or cytokine secretion, in cell-cell communication with cardiomyocytes and other cell types [13]. Under pathological conditions, cardiac fibroblasts are intimately associated with the innate immune system, promoting an adequate response to eliminate insults synergistically [14]. Endotoxins and inflammatory mediators could activate cardiac fibroblasts through toll-like receptors (TLRs), provoking the transcription and release of chemokines and cytokines, such as TNF-α and IL-1β [15]. The chemokines and cytokines released by cardiac fibroblasts could further recruit inflammatory cells from the circulation, amplifying the inflammatory response and releasing abundant myocardial depressant substances in the heart locally to induce cardiac dysfunction [16, 17], suggesting that inhibition of the inflammatory response in cardiac fibroblasts could be a potential therapy for SIC.

Rolipram is a phosphodiesterase type IV specific inhibitor, which could reduce the breakdown of cAMP, and it was primally studied as an antidepressant [18, 19]. Further studies discovered its anti-inflammatory actions. It has been approved by the Food and Drug Administration of the USA (FDA) as a therapeutic drug for chronic obstructive pulmonary disease due to its anti-inflammatory effects [20]. One mechanism of its anti-inflammatory effects is increasing the expression and activity of dual specificity phosphatase 1 (DUSP1), an endogenous negative regulator in infection. Since this enzyme could dephosphorylate both phosphothreonine and phosphotyrosine residues on activated mitogen-activated protein kinases (MAPKs), it was named as dual specificity phosphatase 1 in mice or MAPK phosphatase 1 (MKP1) in humans. It can dephosphorylate p38 MAPK and c-Jun N-terminal kinase (JNK) to inactivate MAPK-mediated inflammatory pathways [21]. A previous study showed that rolipram could reduce the *Staphylococcus aureus*-induced inflammatory responses in macrophages [22]. However, whether its anti-inflammatory effect could be beneficial to cardiac protection in sepsis is still unknown. In the present study, the effects of rolipram on cardiac function were evaluated both in lipopolysaccharide (LPS)-stimulated cardiac fibroblasts in vitro and in endotoxic mice in vivo.

## Methods

### Animals and reagents

C57BL/6 mice were purchased from Southern Medical University. All animal procedures were approved by the Animal Care and Use Committee of Southern Medical University (the permit number 2017 J019), and all animal experiments were conducted in accordance with the Guidelines for Animal Care of Southern Medical University. LPS (L2630) and rolipram (R6520) were purchased from Sigma-Aldrich. Liberase TH Research Grade (0540115001), the enzyme used to digest heart tissues, was purchased from Roche (Mannheim, Germany). The RNA extraction reagent TRIzol was purchased from Life Technologies (Shanghai, China). Reverse transcription kits (FSQ-101) and Real-time PCR Master Mix (QPK-201) were purchased from Toyobo (Osaka, Japan). Enzyme-linked immunosorbent assay (ELISA) kits for TNF-ɑ (88–7324-22), IL-6 (88–7064-86) and IL-1β (88–7013-86) were purchased from Thermo Fisher Scientific (San Diego, CA, USA). The DUSP1 antibody (sc-373,841) was purchased from Santa Cruz (Dallas, Texas, USA), and GAPDH (#2118) was purchased from Cell Signaling Technology (Danvers, MA, USA). Secondary antibody horseradish peroxidase (HRP) conjugated goat anti-rabbit IgG (abs20040) and goat anti-mouse IgG (abs20039) were purchased from Absin (Shanghai, China). Fluorochrome 7-AAD (00–6993-50) was purchased from ThermoFisher (USA), and antibodies specific to Cd45-FITC (553080) and Ly6g-APC-Cy7 (560600) were purchased from BD Biosciences (San Jose, CA, USA).

### Isolation and treatment of neonatal mouse cardiac fibroblasts

Neonatal mouse cardiac fibroblasts were isolated from newborn C57BL/6 mice. Mice were anesthetized with overdose pentobarbital (100 mg/kg) by intraperitoneal injection, and cardiac palpation was used to confirm the death of mice. Then, the hearts of neonatal mice were isolated and cut into pieces. Then, the heart tissues were rinsed with phosphate-buffered saline (PBS) and digested with an enzyme (Liberase TH Research Grade) at 37 °C for 15 min. The supernatant containing the cells was transferred to complete medium (DMEM containing 10% fetal calf serum) to terminate the digestion. Residual tissues were digested with the enzyme at 37 °C for 15 min for further digestion. The digestion was repeated 3 to 4 times to isolate all cells from the heart tissues. All cell suspensions were collected and centrifuged at 250×g for 5 min. The supernatant was discarded, and the precipitate was resuspended in complete medium. The cell suspension was transferred to a culture dish and cultured for 60 min in an incubator at 37 °C and 5% CO_2_. After the unattached cells were discarded, the remaining attached cells were cardiac fibroblasts. After 24 h of cell culture, cardiac fibroblasts were treated with 1 μg/ml LPS for 6 h or 10 μmol/L rolipram for 1 h before LPS stimulation either alone or in combination according to previous studies [23, 24]. Because LPS was dissolved in normal saline (NS), and rolipram was dissolved in dimethylsulfoxide (DMSO), NS and DMSO were used as the solvent control.

### Preparation and treatment of the endotoxemic mouse model

Twenty C57BL/6 mice, male, aged 8 to 10 weeks, were randomly divided into 4 groups (*n* = 5). The endotoxemia mouse model was established by intraperitoneal injection of 15 mg/kg LPS for 6 h according our previous study [24], and 10 mg/kg rolipram [25] was intraperitoneally injected 1 h before LPS injection. Similar with the cell treatment, NS and DMSO were used as the solvent vehicle.

### RNA extraction and quantitative rt-PCR (qRT-PCR)

Total RNA was isolated from cardiac fibroblasts or cardiac tissues with TRIzol reagent. RNA extraction and qRT-PCR were performed as previously described [26]. The primer sequences of TNF-ɑ, IL-6, IL-1β, DUSP1 and GAPDH are shown in the Table 1. The expression results were calculated by the 2^−ΔΔCq^ method reported by Livak and Schmittgen [27].

**Table 1:** The sequences of the rt-PCR primers of TNF-ɑ, IL-6, IL-1β, DUSP1 and GAPDH

Target mRNA	Sequence
TNF-ɑ	
Forward	GATCGGTCCCCAAAGGGATG
Reverse	GGCTTGTCACTCGAATTTTGAGAA
IL-6	
Forward	TCTCTGGGAAATCGTGGAAATGA
Reverse	GACCAGAGGAAATTTTCAATAGGCA
IL-1β	
Forward	AAGCTCTCCACCTCAATGGAC
Reverse	TTGCTTGGGATCCACACTCTC
DUSP1	
Forward	CAGGAAGGACAGGATCTCCA
Reverse	CTGTGCAGCAAACAGTCCAC
GAPDH	
Forward	CTTCAACAGCAACTCCCACTCTTCC
Reverse	GGTGGTCCAGGGTTTCTTACTCC

### Protein extraction

Protein samples were extracted from cultured cardiac fibroblasts or cardiac tissues with lysis buffer (P0013B, Beyotime). For the cultured cardiac fibroblasts, culture medium was removed and washed by cold PBS for three times. Then, cells were lysed by lysis buffer for 20 min on the ice. Then, lysed cells were collected and centrifuged at 12,000 x g for 15 min to pellet debris and the supernatants were used to following measurement. Heart tissue proteins were collected by supersonic splitting. The frequency of sonicator probe was 20 kHz. Tissues were put in a 1.5 mL microcentrifuge tube with 300 μl lysis buffer and gently moved under the tip of the sonnicator probe for 10 s vibrating. Then, incubated on ice for 15 min after the sonication. Then the lysed tissues were centrifuged at 12,000 x g for 20 min and the supernatants were collected for protein analysis. The protein concentrations were quantified by BCA protein assay (ThermoFisher, USA), and same total protein were used to the enzyme immunoassays and western blot.

### Enzyme immunoassay

Enzyme immunoassays for TNF-ɑ, IL-6 and IL-1β in cellular supernatant and heart tissue were carried out according to the manufacturer’s guide. A 96-well plate was coated with the capture antibody of TNF-ɑ (1:250 dilution), IL-6 (1:250 dilution) and IL-1β (1:250 dilution) respectively, incubated overnight at 4 °C and blocked with dilution buffer for 1 h. Then, the standard samples and experimental samples were added and incubated for another 2 h at room temperature. Corresponding detection antibody of TNF-ɑ (1:250 dilution), IL-6 (1:250 dilution) and IL-1β (1:250 dilution), avidin-horseradish peroxidase and tetramethylbenzidine (TMB) solution were added. Lastly, 1 mol/L H_3_PO_4_ was used to stop the reaction. The plate was read at 450 nm, and the concentrations of each sample were calculated according to the standard curve.

### Western blotting analysis

Western blotting analysis was carried out with a procedure described previously [28]. Briefly, equal amounts of protein were subjected to polyacrylamide gel electrophoresis for separation and transferred onto a polyvinylidene fluoride membrane. Protein expression was determined by specific antibodies against DUSP1 (1:1000 dilution) and GAPDH (1:1000 dilution), which diluted in Tris-Buffered Saline Tween-20 (TBST). HRP conjugated anti-mouse IgG (1:5000 dilution) and anti-rabbit IgG (1:10000 dilution) was used as secondary antibody for DUSP1 and GAPDH, respectively. Signals were detected by chemiluminescence and quantified by densitometry.

### RNA interference (RNAi) for DUSP1 knock down

SiRNA (small interfering RNA) that targeted DUSP1 was synthesized by the GenePharma Company (Shanghai, China). The synthetic oligonucleotide sequences of siRNA-DUSP1 and siRNA-NC sequence (as the control) were shown in Table 2. After 24 h isolation, cardiac fibroblasts were incubated in serum-free DMEM with siRNA/Lipofectamine (Life technology, USA) for 6 h, followed by normal culture for 48 h, then cells were pretreated with 10 μmol/L rolipram for 1 h before LPS stimulation. The expression of DUSP1 and the TNF-ɑ, IL-6 concentrations in the supernatant of cardiac fibroblasts with LPS treatment for 6 h were examined with western blot and ELISA methods, respectively.

**Table 2:** The sequences of small interfering RNA

siRNA NC	Sense: CAA UUG UCC UAA CCA CUU UTT
	Antisense: AAA GUG GUU AGG ACA AUU GTT
siRNA DUSP1	Sense: UUC UCC GAA CGU GUC ACG UTT
	Antisense: ACG UGA CAC GUU CGG AGA ATT

### Echocardiographic measurements

Echocardiography was used to measure cardiac function in mice as in our previous study [29]. Animals were lightly anesthetized with 1% inhalant isoflurane and imaged using a 40-MHz linear array transducer attached to a preclinical ultrasound system (SonoScape, Shenzhen, Guangdong, China). M-mode in left ventricular short axis view was used to assess cardiac function by left ventricular ejection fraction (EF).

### Histologic examination

The mice were anesthetized with 50 mg/kg pentobarbital by intraperitoneal injection. The hearts were excised and fixed in 4% paraformaldehyde, then embedded in paraffin. Sections with 4 μm thickness were stained with hematoxylin and eosin for microscopic examination (Nikon, Japan) at a magnification of 200×. The degree of inflammatory infiltration was scored from 1 to 4 according the method reported by Neu et al [30]: Score 1, no inflammatory cell infiltration; 2, the inflammatory infiltration area was less than 10%; 3, inflammatory infiltration area between 10 and 20%; and 4, inflammatory infiltration area over 20%.

### Mouse heart digestion and flow cytometry analysis

Mouse heart cells flow cytometry analysis were processed according to the method described by Chen et al [31]. The mice were anesthetized with 50 mg/kg pentobarbital. Heart tissues were cut into pieces and washed by cold PBS for three times to remove the blood cells. Then the tissues were digested by the enzyme (Liberase TH Research Grade) at 37 °C for 15 min. The supernatant containing the cells was transferred to the medium with 10% fetal calf serum to terminate the digestion. Residual tissues were digested with the enzyme at 37 °C for 15 min for further digestion. The digestion was repeated 3 to 4 times to isolate all cells from the heart tissues. All cell suspensions were collected and centrifuged at 250×g for 5 min. The supernatant was discarded, and the precipitate was resuspended in PBS and cells were filtered through a 70 μm nylon mesh (ThermoFisher, USA). Erythrocytes were removed by RBC lysis Buffer (ThermoFisher, USA). Cells were stained by 7-AAD, antibodies specific to Cd45-FITC and Ly6g-APC-Cy7 for 30 min. The cells were washed twice by cold PBS and subjected to flow cytometry (BD FACSCalibur) measurement. Data were analysed by FlowJo. 7-AAD negative cells were regarded as the alive cells. Leukocyte cell ratio was calculated by Cd45 positive cells/all alive cells. Neutrophil ratio was calculated by Ly6g positive cells/Cd45 positive cells.

### Statistical analysis

All data were analyzed by SPSS 13.0 software (IBM, Armonk, New York, USA). After the homogeneity of variance test, one-way ANOVA followed by the Newman-Keuls test was performed for multigroup comparisons in the LPS-stimulated cardiac fibroblasts experiment. Two-way ANOVA was performed to evaluate the effect of rolipram both in cardiac fibroblasts and endotoxemic mice. A value of *P* < 0.05 was considered indicative of statistical significance.

## Results

### LPS increased the expression and secretion of inflammatory cytokines in cardiac fibroblasts

**Fig. 1:**
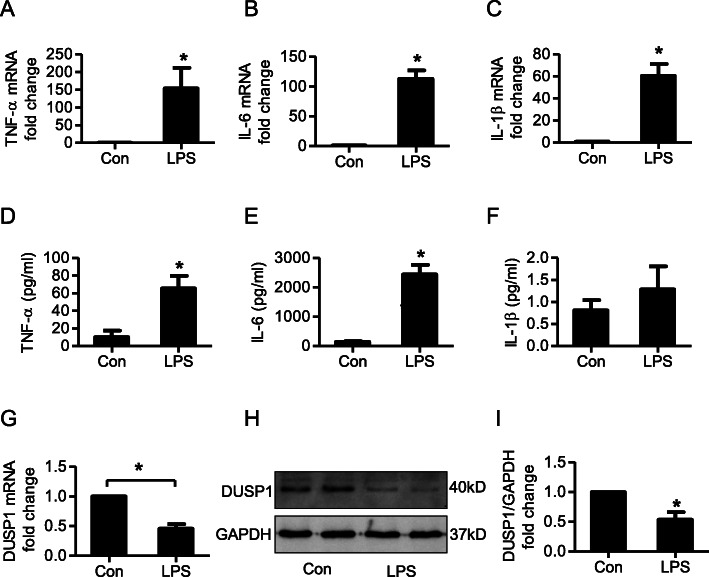
Effects of LPS on the cardiac fibroblast inflammatory responses and DUSP1 expression. After cardiac fibroblasts were stimulated with 1 μg/ml LPS for 6 h, the mRNA levels and supernatant concentrations of inflammatory mediators were measured by qRT-PCR and ELISA, respectively. **a-c** and **g**, Relative fold change to control group of TNF-ɑ, IL-6, IL-1β and DUSP1 mRNA expression. **d-f**, Concentrations of TNF-ɑ, IL-6 and IL-1β in the supernatant. **h**, DUSP1 protein expression in cardiac fibroblasts (original, uncropped blot was shown in supplement Fig. S1A). I, Protein quantification of DUSP1. * represents *P* < 0.05 compared with the control group

After cardiac fibroblasts were stimulated with 1 μg/ml LPS for 6 h, the mRNA levels and supernatant concentrations of inflammatory mediators were measured. As shown in Fig. 1, the mRNA levels of TNF-ɑ, IL-6 and IL-1β were greatly upregulated, and the concentrations of TNF-ɑ and IL-6 in supernatant also increased significantly after LPS stimulation (Fig. 1d and e). However, although the mRNA expression of IL-1β increased considerably after LPS stimulation, no significant difference or even a moderate increase was observed in IL-1β concentration in supernatant (Fig. 1f).

### Rolipram inhibited the LPS-induced inflammatory response by upregulating DUSP1 expression in cardiac fibroblasts

Cardiac fibroblasts were treated with 1 μg/ml LPS for 6 h or 10 μmol/L rolipram for 1 h before LPS stimulation either alone or in combination. The mRNA levels (Fig. 1g) and protein expression (Fig. 1h) of DUSP1 in cardiac fibroblasts were measured 6 h after LPS stimuli.

DUSP1 is a negative modulatory factor in the inflammatory response and may be upstream of LPS-induced proinflammatory cytokine release in cardiac fibroblasts. Therefore, in our study, the expression of DUSP1 was measured by qRT-PCR and western blotting after LPS treatment, and our results showed that both the mRNA (Fig. 1g) and protein expression (Fig. 1h and i) of DUSP1 were decreased significantly after LPS stimulation (Fig. 1 g-i), indicating that the downregulation of DUSP1 might participate in the LPS-induced inflammatory response in cardiac fibroblasts.

**Fig. 2:**
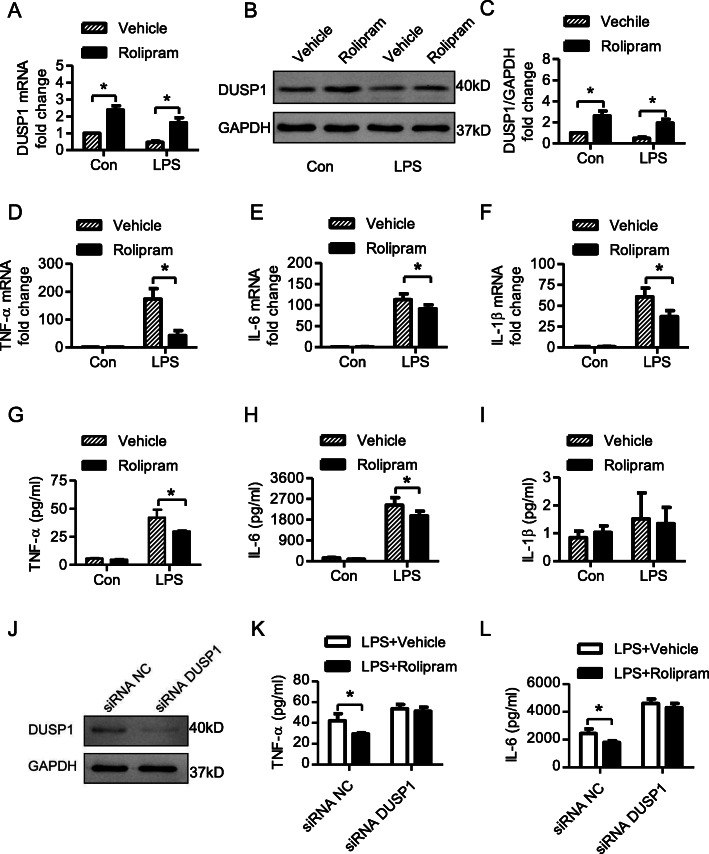
Effects of rolipram on the LPS-induced DUSP1 expression and inflammatory response in cardiac fibroblasts. Cells were pretreated with 10 μmol/L rolipram for 1 h before LPS stimulation. DUSP1 mRNA and protein expression, the mRNA expression and supernatant protein concentrations of inflammatory mediators in cardiac fibroblasts were measured after LPS stimuli for 6 h. For the RNAi experiment, cardiac fibroblasts were treated by small interfering RNA for 6 h, followed by normal culture for 48 h, then cells were pretreated with 10 μmol/L rolipram for 1 h before LPS stimulation. The expression of DUSP1 and the TNF-ɑ, IL-6 concentrations in the supernatant of cardiac fibroblasts were measured. **a**, Relative fold of changes to control of DUSP1 mRNA; **b** and **c**, DUSP1 expression detection and quantification (original, uncropped blot was shown in supplement Fig. S1B). **d-f**, Relative fold of changes to control of TNF-ɑ, IL-6 and IL-1β mRNA expression. **g-i**, Concentrations of TNF-ɑ, IL-6 and IL-1β in the supernatant. **j**. The expression of DUSP1 after RNAi (original, uncropped blot was shown in supplement Fig. S1C). **k** and **l**. The TNF-ɑ and IL-6 concentrations in the supernatant after the DUSP1 knockdown* represents *P* < 0.05

A previous study found that rolipram could upregulate the expression of DUSP1 in macrophages [22]. To investigate its effect on the LPS-induced inflammatory response in cardiac fibroblasts, rolipram was used before LPS administration. As shown in Fig. 2, pretreatment with 10 μmol/L rolipram significantly reversed the LPS-induced downregulation of DUSP1 at both the mRNA and protein levels (Fig. 2a-c).

Rolipram significantly inhibited LPS-induced upregulation and secretion of TNF-α (Fig. 2d and g) and IL-6 (Fig. 2e and h) in cardiac fibroblasts but not IL-1β secretion (Fig. 2i). After knocking down the expression of DUSP1 by targeted siRNA, rolipram has no effect on the inflammatory cytokine secretion (Fig. 2j-l), indicating that the protective effect of rolipram on inflammatory response was dependent on the DUSP1 expression.

### Rolipram inhibited the LPS-induced inflammatory mediator release by upregulating DUSP1 expression in the hearts of endotoxic mice

**Fig. 3:**
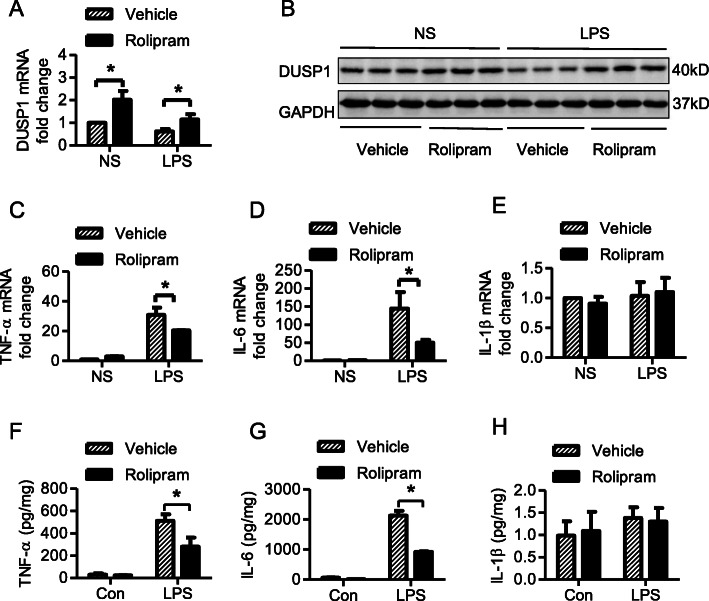
Effect of rolipram on the inflammatory response in the hearts of endotoxic mice. Endotoxic mice were established by intraperitoneal injection of 15 mg/kg LPS for 6 h. In the rolipram group, 10 mg/kg rolipram was injected intraperitoneally 1 h before LPS injection. The hearts of the mice were dissociated, and the mRNA and protein levels of proinflammatory molecules (TNF-ɑ, IL-6 and IL-1β) and DUSP1 were measured by qRT-PCR and western blotting assays. **a**, Relative fold of changes to control of DUSP1 mRNA expression in heart tissues. **b**, Representative image of western blots of DUSP1 expression (original blot was shown in supplement Fig. S1D). **c-e**, Relative fold of changes to control of TNF-ɑ, IL-6 and IL-1β mRNA expression in heart tissues. **f-h**, The concentration of TNF-ɑ, IL-6 and IL-1β in heart tissues. * represents *P* < 0.05, *n* = 5

To further evaluate the likely role of rolipram in vivo, we established an endotoxic mouse model by intraperitoneal injection of 15 mg/kg LPS for 6 h. In the LPS + rolipram group, 10 mg/kg rolipram was injected intraperitoneally 1 h before LPS injection. The hearts of the mice were dissociated, and the mRNA and protein levels of the proinflammatory molecules and DUSP1 were measured. Similar with the in vitro results, LPS treatment significantly decreased the mRNA levels of DUSP1 in the heart (Fig. 3a). In addition, rolipram could increase the expression of DUSP1 at both the mRNA and protein levels (Fig. 3a and b), similar with the results in cardiac fibroblasts. The mRNA levels of TNF-α and IL-6 were decreased in the LPS + rolipram group compared with those in the LPS + vehicle group (Fig. 3c and d). However, no change in IL-1β mRNA was observed in the hearts of endotoxic mice (Fig. 3e), in contrast with the cultured cardiac fibroblasts. The protein levels of TNF-α, IL-6 and IL-1β in heart tissue were also measured by ELISA. TNF-α and IL-6 levels were increased significantly in the LPS group compared with those in the control group, and rolipram pretreatment decreased the LPS-induced production of TNF-α and IL-6 (Fig. 3f and g).

### Rolipram reduced the leukocyte infiltration and improved heart dysfunction in endotoxic mice

**Fig. 4:**
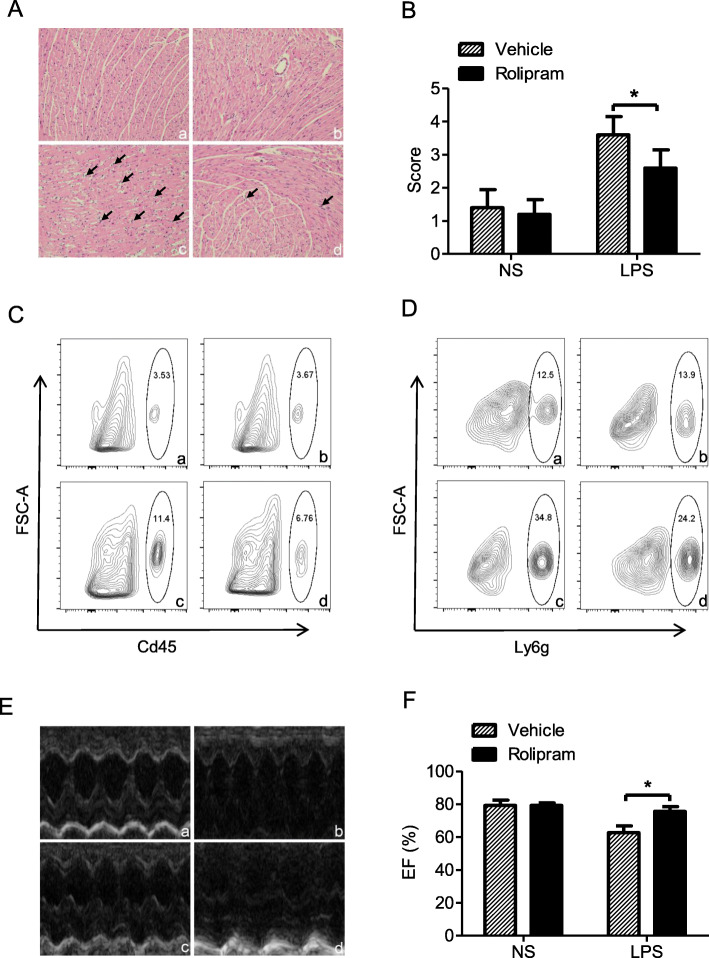
Effects of rolipram on inflammatory cell infiltration and cardiac function of the hearts of endotoxic mice. Inflammatory cell infiltration and heart function were examined on the endotoxic mice model by pathological analysis, flow cytometry and echocardiography, respectively. **a**. Representative pictures of HE staining of heart tissue from endotoxic mice (200×, the arrows showed the inflammatory cells). **b**. The inflammatory scores of the histopathological sections. **c**. The Cd45 positive cell rate in the single cells harvested from the heart tissue. **d**. The Ly6g positive cell rate in the Cd45 positive cells harvested from the heart tissue. **e**. Representative pictures of M-mode at the level of left ventricular short axis view. **f**. Analysis of left ventricular ejection fraction (EF) of the hearts of endotoxic mice. **a**, control + DMSO, **b**, control + rolipram, **c**, LPS + DMSO, **d**, LPS + rolipram. * represents *P* < 0.05, *n* = 5

Inflammatory cell infiltration was an important indicator of the tissue inflammatory response, and our pathological results showed that inflammatory cell infiltration was present in the myocardial interstitium. However, the degree of inflammatory cell infiltration in the rolipram pretreatment group was significantly reduced (Fig. 4a). The inflammatory infiltration scores were also lower in the LPS + rolipram group than in the LPS + vehicle group (Fig. 4b). In addition, flow cytometry analysis showed that rolipram treatment could decrease the leukocyte ratios (Fig. 4c) and neutrophil ratios (Fig. 4d) in endotoxic mice heart. These results suggest that rolipram may inhibit the LPS-induced inflammatory response by upregulating DUSP1 expression in the hearts of endotoxic mice.

Finally, echocardiography was used to measure the effects of rolipram on endotoxin-induced cardiac dysfunction in mice. M-mode at the level of the papillary muscles was used to assess changes in left ventricular EF. Representative M-mode images of the left ventricular short axis view are shown in Fig. 4e. Compared with the NS + vehicle group, the EF was decreased in the LPS + vehicle group, suggesting that LPS could induce cardiac dysfunction. However, EF was higher in the LPS + rolipram group than in the LPS + vehicle group, indicating that rolipram may improve the heart dysfunction induced by endotoxin.

## Discussion

The present in vitro and in vivo study showed that LPS could induce inflammatory responses in cardiac fibroblasts with high levels of expression and secretion of inflammatory cytokines (TNF-α, IL-6, IL-1β) and could increase inflammatory cell infiltration in heart tissues, further inducing heart dysfunction, or SIC, in endotoxic mice. Moreover, LPS could also decrease the transcription and expression of DUSP1 (an endogenous negative regulator that inactivates MAPK-mediated inflammatory pathways) in cardiac fibroblasts and heart tissue. However, pretreatment with rolipram (an anti-inflammatory drug that selectively increases DUSP1 expression) could significantly reverse LPS-induced downregulation of DUSP1 and inhibit LPS-induced upregulation and secretion of TNF-α and IL-6 to further reduce neutrophil cell infiltration and improve heart dysfunction induced by endotoxin in mice.

Sepsis is a syndrome of physiologic, pathologic, and biochemical abnormalities induced by infection, which may have originated as pneumonia, peritonitis, urinary system infection, and the like [1]. Overall, 40–50% of cases of sepsis are complicated by SIC, and the mortality of the patients with SIC can exceed 50% [32]. However, the exact mechanism of SIC remains unclear. One explanation for SIC is the systemic inflammatory response. Since the viewpoint of “circulating myocardial depressant factor” was put forward, an increasing number of researchers have found that endotoxin and inflammatory mediators affect cardiac depression [33]. In sepsis, SIC was more common in Gram-negative infection induced endotoxemia than that in Gram-positive infection, for the exotoxin from Gram-positive bacteria usually resulted in local symptom [34, 35]. Therefore, we established an endotoxemia model by LPS treatment. In our study, we found that the concentrations of TNF-ɑ and IL-6 were significantly increased in the heart tissue of endotoxic mice, which might be involved in the occurrence of cardiac dysfunction.

Heart cell components include cardiomyocyte, fibroblast, immune cell, endothelial cell, stromal cell [36] . Inflammatory responses not only existed in the immune cells, but also the cardiac cells. Yao et al found that constitutive activation of inflammasome were found in cardiomyocyte, which contributed to the atrial fibrillation development [37]. Here, we found that LPS could activate cultured cardiac fibroblasts and increase the transcription of TNF-ɑ and IL-6. These inflammatory mediators might act as myocardial depressant substances in the process of SIC. In addition, the chemokines and cytokines released by cardiac fibroblasts could further recruit inflammatory cells in the circulation, amplifying the inflammatory response and the level of abundant myocardial depressant substances locally in the heart. By HE staining, we also found significant inflammatory cell infiltration in cardiac pathological sections. However, though the mRNA of IL-1β was increased in cultured cardiac fibroblasts after LPS stimulation, we did not find that IL-1β protein increased either in vitro or in vivo. This finding might be due to the immaturity of IL-1β. The secretion of IL-1β occurs only after the precursor protein of IL-1β is cleaved, for which inflammasome activation is required [38], although LPS stimulation alone was not enough to activate the inflammasome.

Rolipram has been proven to have anti-inflammatory effects on chronic obstructive pulmonary disease. Recently, rolipram was found to decrease the expression of proinflammatory cytokines and mortality of sepsis animals [39]. Our results showed that rolipram could inhibit the transcription and release of TNF-ɑ and IL-6 in cardiac fibroblasts, indicating that rolipram has an anti-inflammatory effect on cardiac cells. An in vivo study showed that rolipram pretreatment could protect against cardiac dysfunction and induced a concomitant decrease in TNF-ɑ and IL-6 mRNA. These results supported that local inflammatory cytokines released by cardiac fibroblasts may be a depressive factor in cardiac dysfunction. In addition, since TNF-ɑ was an exogenous signal which could promote cell death by activating caspase-8, rolipram pretreatment might prevent cardiomyocytes from TNF-ɑ mediated cell death. This could be another potential protective mechanism of the rolipram.

The anti-inflammatory effects of rolipram might be associated with a concomitant increase in the expression of DUSP1 [40]. Similarly, we also found that rolipram could increase the expression of DUSP1 in cardiac fibroblasts by affecting the transcriptional process, as evidenced by the increased DUSP1 mRNA levels both in vitro and in vivo. DUSP1, the first identified molecule that could dephosphorylate MAPKs, could impede the cellular functions of MAPKs [41]. By deactivating MAPKs, DUSPs can modulate both innate and adaptive immunity. A previous study found that DUSP1-deficient bone marrow-derived macrophages showed prolonged activation of p38 MAPK and JNK, leading to increased cytokine production [42]. Activated MAPKs could promote the translocation of transcription factors, such as nuclear factor-κB (NF-κB) and activator protein 1 (AP-1), potentially increasing the transcription of inflammatory mediators [43]. Consistent with this notion, administering intraperitoneal injections of LPS to DUSP1-deficient mice caused increased lethality and enhanced production of IL-6 and TNF-ɑ [44]. Previous results showed that DUSP1 was an important negative feedback molecule limiting the proinflammatory response in the heart during sepsis [45]. Our results showed that the expression of DUSP1 was decreased at 6 h after LPS stimulation. The lack of a negative feedback molecule might lead to an inflammatory response. These results indicated that targeting DUSP1 may be beneficial for limiting the inflammatory response in the heart under septic conditions. Rolipram may act on the transcription factor for DUSP1 and promote transcription. However, the exact mechanism of this upregulation is unknown, and further study is needed.

In the present study, HE staining of cardiac pathological sections and flow cytometry analysis showed that rolipram treatment could reduce infiltration of leukocytes in the endotoxemia mice. These results remind us to speculate that that rolipram might reduce chemokines locally in the heart. Kim et al found that DUSP1 deficiency results in increased monocyte adhesion and migration and increased monocyte chemoattractant protein-1 (MCP-1) in macrophages [46]. Since we found that rolipram could upregulate the expression of DUSP1 in cardiac fibroblasts, we speculate that the effect of rolipram on chemoattractants may be related to the upregulation of DUSP1. In addition, rolipram has been found that rolipram could hydrolyzes the second messenger cyclic adenosine monophosphate (cAMP), which is a key regulator of many important physiological processes [22]. This might be another explanation of the effects of rolipram on the chemoattractants, but further convincing evidence is required and more experiments will be carried out to clarify this hypothesis.

Though our findings are encouraging, still more studies are needed before rolipram could be employed in clinical for treating sepsis-cardiomyopathy. Here are the limitations of the study: 1) present study focused on the effect of rolipram on the cardia fibroblasts, and its effect on cardiomyocytes was not clear; 2) we found rolipram treatment rescued the decreased ejection fraction in endotoxic mice, but the mechanism of this rescue remains unclear (i.e. whether rolipram regulates myocardial contractility, cardiomyocyte viability, etc.); 3) HE staining of cardiac pathological sections and flow cytometry analysis showed that rolipram treatment could reduce infiltration of leukocytes in the endotoxic mice, but additional experiments are required to examine for rolipram’s effects on chemoattractants.

## Conclusions

Our results indicated that rolipram could protect against LPS-induced cardiac injury by inhibiting the inflammatory response in cardiac fibroblasts and that this effect may be associated with the upregulation of DUSP1. Rolipram could be a potential therapy for sepsis-induced cardiomyopathy by mediating the inflammatory response in cardiac fibroblasts.

## Supplementary information

**Additional file 1: Figure S1.** Original and uncropped blots of all western blot. A. Original blots of the western blot shown in Fig. 1h. B. Original blots of the western blot shown in Fig. 2b. C. Original blots of the western blot shown in Fig. 2j. D. Original blots of the western blot shown in Fig. 3b.

## Data Availability

The data and material used to support the findings of this study are available from the corresponding author upon request.

## Abbreviations

cAMP: Cyclic adenosine monophosphate
DMSO: Dimethylsulfoxide
DUSP1: Dual specificity phosphatase 1
EF: Ejection fraction
ELISA: Enzyme-linked immunosorbent assay
IL-1β: Interleukin-1β
IL-6: Interleukin-6
JNK: C-Jun N- terminal kinase
LPS: Lipopolysaccharide
MDF: Myocardial depressant factor
MAPKs: Mitogen-activated protein kinases
MCP-1: Monocyte chemoattractant protein-1
MKP1: MAPK phosphatase 1
NF-κB: Nuclear factor-κB
NS: Normal saline
rt-PCR: Real-time PCR
SIC: Sepsis-induced cardiomyopathy
SiRNA: Small interfering RNA
TLRs: Toll-like receptors
TMB: Tetramethylbenzidine
TNF-α: Tumor necrosis fator-α
