# Supplementary material for: Protective effects of rolipram on endotoxic cardiac dysfunction via inhibition of the inflammatory response in cardiac fibroblasts
Source: BMC Cardiovasc Disord. 2020 May 24;20:242. doi: 10.1186/s12872-020-01529-7 (PMC7247226; doi:10.1186/s12872-020-01529-7)
Supplement: Supplementary file 1 — Additional file 1: Figure S1. Original and uncropped blots of all western blot. A. Original blots of the western blot shown in Fig. 1h. B. Original blots of the western blot shown in Fig. 2b. C. Original blots of the western blot shown in Fig. 2j. D. Original blots of the western blot shown in Fig. 3b. [file 12872_2020_1529_MOESM1_ESM.ppt]

## Slide 1
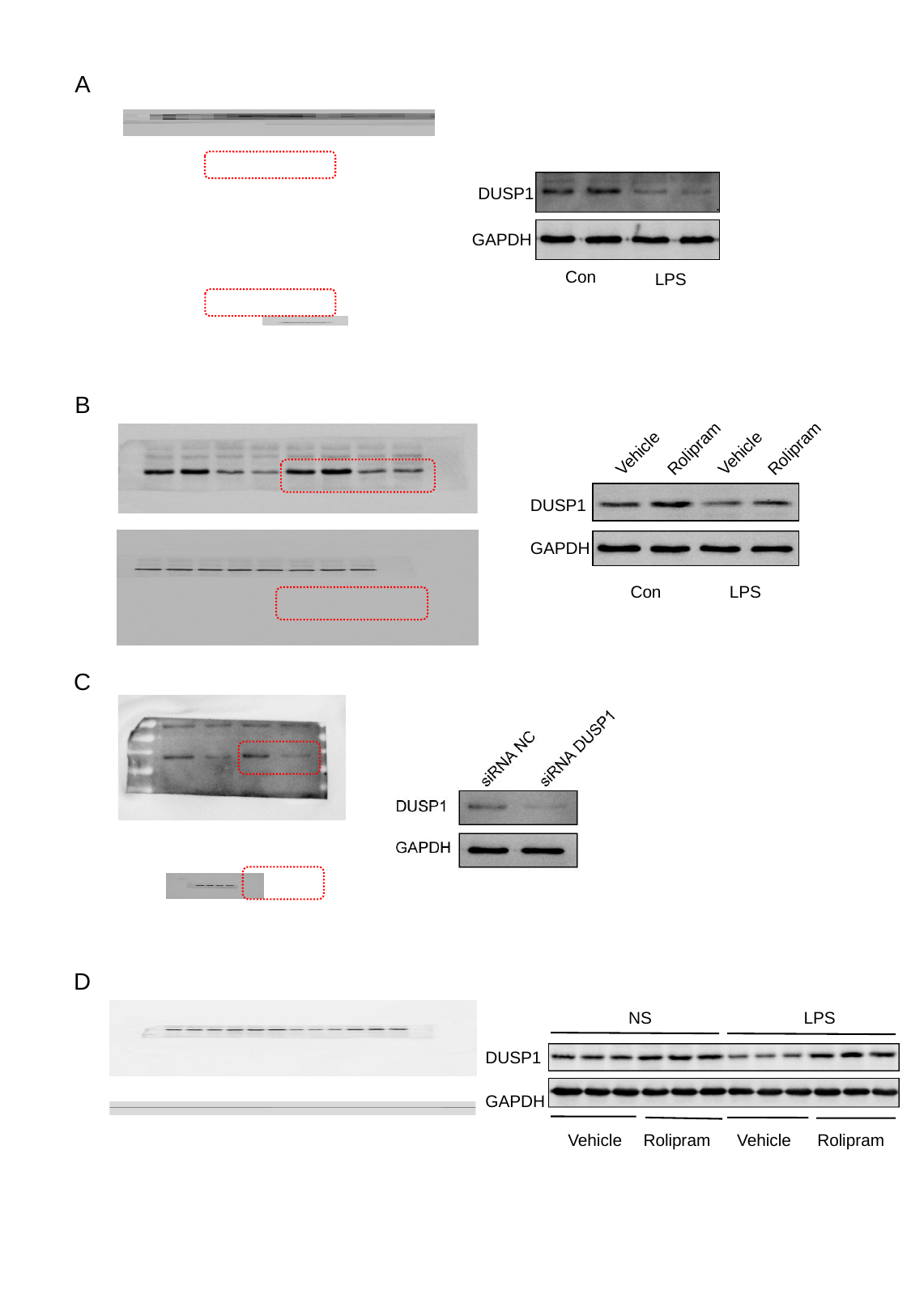

A
DUSP1
GAPDH
Con
LPS
B
Rolipram
Rolipram
Vehicle
Vehicle
DUSP1
GAPDH
Con
LPS
C
D
NS
LPS
DUSP1
GAPDH
Vehicle
Rolipram
Vehicle
Rolipram
